# Functional Analysis of Conserved Transmembrane Charged Residues and a Yeast Specific Extracellular Loop of the Plasma Membrane Na^+^/H^+^ Antiporter of *Schizosaccharomyces pombe*

**DOI:** 10.1038/s41598-019-42658-0

**Published:** 2019-04-17

**Authors:** Debajyoti Dutta, Asad Ullah, Sana Bibi, Larry Fliegel

**Affiliations:** Department of Biochemistry, University of Alberta, Edmonton, Alberta T6G 2H7 Canada

**Keywords:** Ion channels, Sodium channels

## Abstract

The Na^+^/H^+^ exchanger of the plasma membrane of *S*. *pombe* (*Sp*NHE1) removes excess intracellular sodium in exchange for an extracellular proton. We examined the functional role of acidic amino acids of a yeast specific periplasmic extracellular loop 6 (EL6) and of Glu^74^ and Arg^77^ of transmembrane segment 3. Glu^74^ and Arg^77^ are conserved in yeast species while Glu^74^ is conserved throughout various phyla. The mutation E74A caused a minor effect, while mutation R77A had a larger effect on the ability of *Sp*NHE1 to confer salt tolerance. Mutation of both residues to Ala or Glu also eliminated the ability to confer salt tolerance. Arg^341^ and Arg^342^ were also necessary for *Sp*NHE1 transport in *S*. *pombe*. Deletion of 3 out of 4 acidic residues (Asp^389^, Glu^390^, Glu^392^, Glu^397^) of EL6 did not greatly affect *Sp*NHE1 function while deletion of all did. Replacement of EL6 with a segment from the plant Na^+^/H^+^ exchanger SOS1 also did not affect function. We suggest that EL6 forms part of a cation coordination sphere, attracting cations for transport but that the region is not highly specific for the location of acidic charges. Overall, we identified a number of polar amino acids important in *Sp*NHE1 function.

## Introduction

Na^+^/H^+^ exchangers are integral membrane proteins that exist in all known plants, yeast and mammalian cells^1^. In mammals, they remove excess intracellular protons in exchange for external sodium and are important in ischemic heart disease and breast cancer^2,3^. In contrast, in plants and yeast, plasma membrane members of this group of proteins catalyze the reverse process: exchanging intracellular sodium for extracellular protons. They therefore function in salt tolerance extruding sodium ions through these plasma membrane transporters. The energy of transport comes from the proton gradient generated by the plasma membrane H^+^-ATPase^4^. In plants, salt tolerance is significant in agriculture and in this regard, overexpression of plasma membrane salt tolerance proteins has been shown to improve salt tolerance in plants^5^.

In the fission yeast *Schizosaccharomyces pombe*, the Na^+^/H^+^ exchanger *Sp*NHE1 (previously known as sod2) plays the major role in salt removal from the cytosol and in salt tolerance. It is an excellent model system with which to study plasma membrane salt tolerance proteins^6^. *S*. *pombe* has limited other salt tolerance mechanisms in the plasma membrane, so the deletion of *Sp*NHE1 causes this yeast to exhibit a salt sensitive phenotype^7^. By reintroducing the protein, salt tolerance is restored and this allows assay of defective protein’s activity. We have used this assay in earlier works^6,8,9^. *Sp*NHE1 can also enhance salt tolerance in plants^10^ and in phylogenetic analysis *Sp*NHE1 clusters with plant plasma membrane salt tolerance proteins^11^.

The mechanism of transport of plasma membrane salt tolerance proteins and Na^+^/H^+^ exchangers in general, is of interest both as a fundamental scientific problem and from the economic view of modification of plants to improve agricultural output. Crystal structures of some forms of Na^+^/H^+^ exchanger are known such the *E*. *coli* sodium transporter NhaA^12^, *Thermos thermophilus* (NapA)^13^, *Pyrococcus abyssi* (PaNhaP)^14^ and *Methanococcus jannaschii* (MjNhaP1)^15^. However, details of the transport mechanism of other kinds of Na^+^/H^+^ exchangers such as *Sp*NHE1 are less clear^6^. We^6^ recently demonstrated that TM XI (amino acids 360–393) of *Sp*NHE1 is critical in transport. It has a helix-extended region-helix like structure that is characteristic of the ion translocation center of Na^+^/H^+^ exchangers^12,16^. However, less is known about neighboring regions and their functions. In this contribution, we studied a yeast specific periplasmic loop (EL6) and acidic residues of putative transmembrane segment 3 and 11. We examined the function of EL6 and determined that it contains several conserved amino acids that together, are critical in function. Our results are the first demonstration of the role of this yeast specific periplasmic loop and characterization of these residues of TM3 and 11.

## Results

### SpNHE1 alignment and modeling

**Figure 1:**
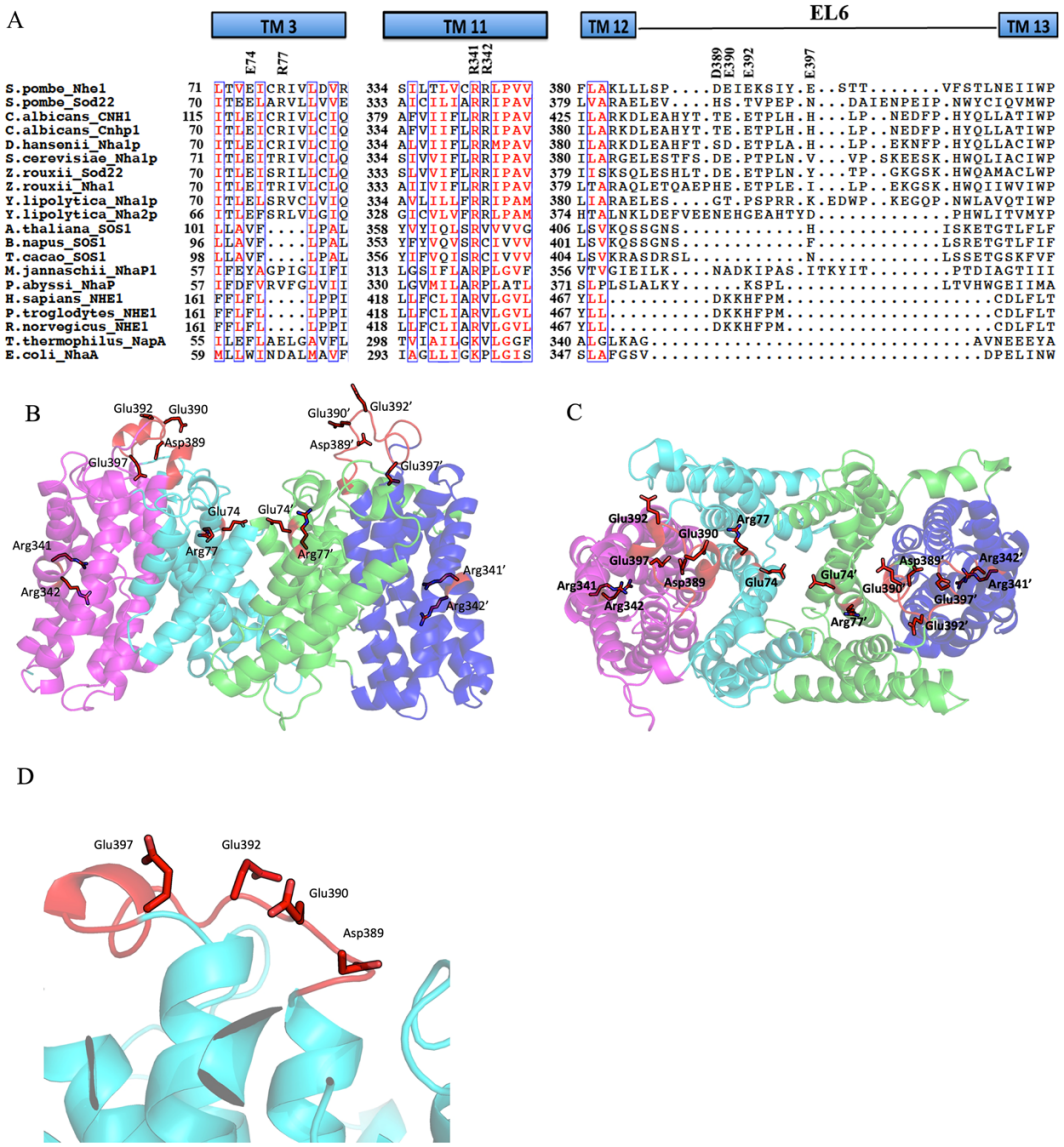
Sequence alignment and molecular model of TM XI and distal region of *Sp*NHE1. (**A**) Alignment of sequences of yeast, fungi, plant, mammalian and bacterial plasma membrane Na^+^/H^+^ exchangers. Figure illustrates intracellular loop 6 (IL6) and TMs 3, 11–13 of *Sp*NHE1. Predicted secondary structures and putative TM 12 of *Sp*NHE1 are highlighted above the sequence alignment. Conserved amino acids are colored red and conserved regions are boxed. Representatives included are; yeast and fungi group *S*. *pombe*NHE1 (**NP_592782**.**1**), *S*. *pombe*Sod22 (**NP_594194**.**1**), *Candida albicans* CNH1 (**XP_710352**.**1**), *C*. *albicans* Cnh1p (**AAL24468**.**1**), *Debaryomyces hansenii* Nha1p (**CAI45290**.**1**), *Saccharomyces cerevisiae* Nha1p (**NP_013239**.**1**), *Zygosaccharomyces rouxii* Sod22 (**XP_002497045**.**1**), *Z*. *rouxii* Nha1 (**XP_002497801**.**1**), *Yarrowia lipolytica* Nha1p (**XP_501299**.**1**), *Y*. *lipolytica* Nha2p (**XP_503447**.**1**). From the plants *Arabidopsis thaliana* SOS1 (**AAL32824**), *Brassica napus* SOS1 (**AGA37213**.**1**), and *Theobroma cacao* SOS1 (**EOY01238**.**1**) are included. *Methanocaldococcus jannaschii* NhaP1 (**NP_247021**.**1**), *Pyrococcus abyssi* NhaP (**CAB50204**) are archaeon NHEs. From mammals, *Homo sapiens* NHE1 (**NP_003038**.**2**), *Pan troglodytes* NHE1 (**XP_016812591**) and *Rattus norvegicus* NHE1 (**AAA98479**) are included. Finally, *Thermus thermophiles* NapA (**YP_144738**), and *Escherichia coli* NhaA(**WP_000681354**) are from the bacterial group. Putative TM segments TM3, TM10, and parts of TM12-TM13 of *Sp*Nhe1 are indicated on top of the sequence alignment. TM12-TM13 joining loop is designated as IL6. Conserved amino acids are coloured red and conserved regions are boxed. Glu74, Arg77, Arg341, Arg342, and the acidic residues Asp389, Glu390, Glu392, and Glu397 of IL6 are shown. The number of the first residue of each TM segment is indicted. (**B**) Molecular model of *Sp*NHE1 from^17^ illustrating side view of two protomers of a dimer seen parallel to the membrane. Protomers are colored magenta or blue for the transport domains, and cyan or green for the dimerization domains. Side chains of extracellular loop 6 Asp389, Glu390, Glu392 and Glu397 are shown in red. Glu74, Arg77, Arg341 and Arg342 side chains are also shown in red. Residues from the second protomer are indicated with “’”. (**C**) Molecular model of *Sp*NHE1 from^17^ illustrated the two protomers as seen perpendicular to the membrane. Colors and side chain labeling are as in “B”. (**D**) Close up view of *Sp*NHE1 from^17^ illustrating EL6 and acidic residues.

We examined amino acids of *Sp*NHE1 in or near putative transmembrane (TM) segments 3, 11 and 12^17^. Figure 1A is an alignment of the sequence of *Sp*NHE1 near these TM segments, with a number of Na^+^/H^+^ exchanger types from a variety of species. Amino acids 334–346 form part of the putative TM XI as described earlier^17^. As noted earlier, this region is highly conserved, especially within yeast species, but even across plants and other organisms. Following this region, there is a sequence of amino acids ^381^LAKLLLSPDEIEKSIYESTTVFSTLN^406^ and within this sequence, the core of amino acids ^389^DEIEKSIYESTT^400^ appear to be present principally in yeast species. Amino acids Asp^389^, Glu^390^, Glu^392^ and Glu^397^ are acidic amino acids (indicated) that are present within this sequence. While the presence of the region is confirmed, there is considerable variation especially in acidic amino acids Asp^389^ and Glu^390^. Glu^390^, and Glu^397^ are more conserved within yeast species. The sequence ^390^EIEKSIYE^397^, roughly corresponded to QSSGNSHIKE of *Arabidopsis thaliana* SOS1.

The TM 11 region (amino acids 329–351) is relatively highly conserved. Arg^341^ (indicated) is conserved across yeast species and across other more distantly related antiporters. Arg^342^ is only conserved across the yeast species. Other regions of hydrophobic amino acids (boxed) show conservation; however, hydrophobic residue substitution does occur in some areas.

Upstream, TM3 contains polar residues Glu^74^ and Arg^77^ that are highly conserved among the yeast species. Some hydrophobic amino acids are conserved, though generally less so than TM 11.

We next examined the putative position of some of the conserved polar residues in our recently published model of *Sp*NHE1^17^. Two protomers of the putative dimer are illustrated (Fig. 1B,C). Amino acids Asp^389^, Glu^390^, Glu^392^ and Glu^397^ were present on an extracellular loop projecting away from the membrane. A close up of these residues (Fig. 1D) illustrates that they are close to one another in a somewhat extended conformation. Residues Glu^74^ and Arg^77^ were present somewhat within the putative membrane bilayer region, and were possibly oriented such that they could be involved in the interface of the two subunits of the dimer. Arg^341^ and Arg^342^ were closer to a mid membrane position.

### Mutant growth and expression

To examine the importance of the amino acids that we identified as being conserved, and possibly important in function, we created a series of *Sp*NHE1 mutations that were expressed in the sod2:ura4 strain. The mutant proteins expressed that had a single amino acid changed were E74A, R77A, R341A, R342A, R341E and R342E and E397L. The mutants with two amino acids changed were E74R77A and E74R77RE. E74R77A had the indicated amino acids both changed to Ala. E74R77RE had the amino acids changed as indicated, switching their charge. Other proteins with more amino acids changed included the NQQ, NQQQ, DEL6 and REL6 mutants. The NQQ mutant had the mutations D389N,E390Q,E392Q to EL6. The NQQQ mutant had the same mutations as the NQQ mutant plus an E397Q mutation. The DEL6 mutant had the amino acids ^390^EIEKSIYE^397^ deleted. The REL6 mutant had the amino acid residues ^390^EIEKSIYE^397^ replaced with SOS1-like residues from the protein SOS1 (QSSGNSHIKE).

**Figure 2:**
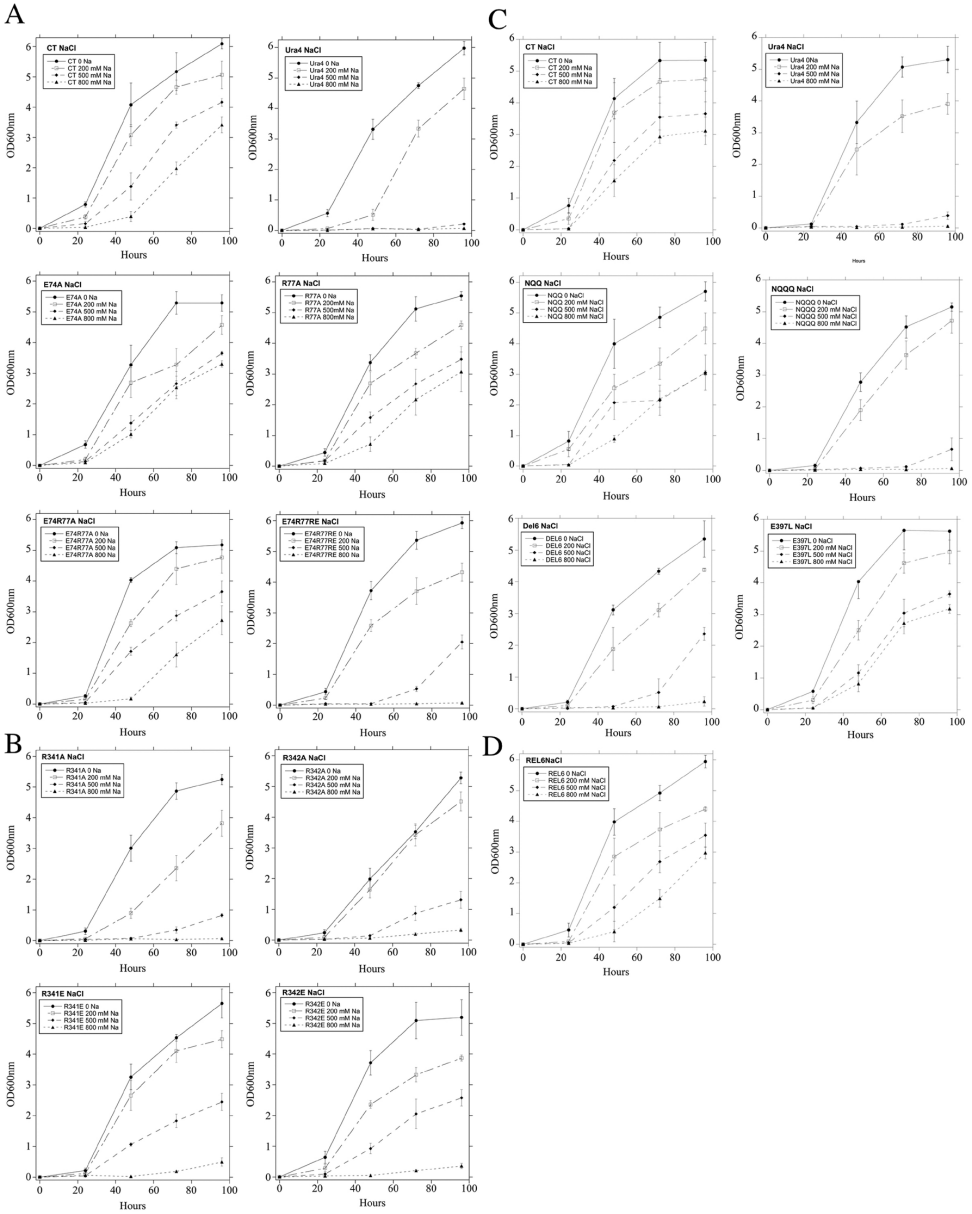
Growth of *S*. *pombe* containing either wild type or *Sp*NHE1 proteins in liquid media with concentrations of NaCl of 0, 200 mM, 500 mM or 1 M NaCl. To assess NaCl tolerance of strains media was inoculated with 2 × 10^6^ cells into 2.5 ml of medium at 30 °C for up to 72 hours. Cell absorbance of suspensions at 600 nm was used to monitor growth at the indicated times. Results are the mean +/− SE of a minimum of three determinations. (**A**,**B**) Comparison of growth rates in NaCl medium of control and mutant strains. (**C**,**D**) Second round of mutagenesis to other amino acids or segments of *Sp*NHE1. Ura4 refers to *S*. *pombe* with the *Sp*NHE1 knockout described earlier^8^.

**Figure 3:**
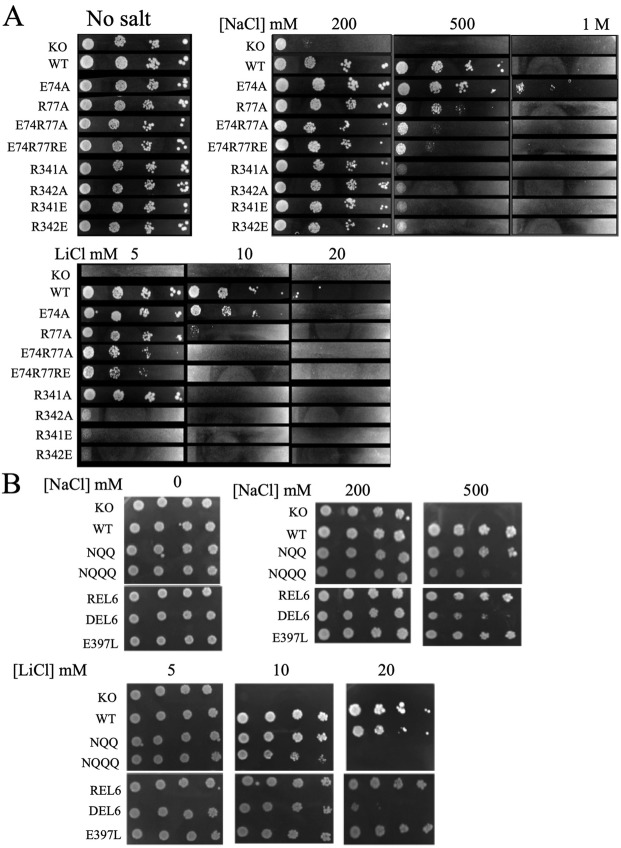
Growth of wild type (WT) and mutant *Sp*NHE1 on solid media in the presence or absence of varying amounts of NaCl or LiCl (**A**,**B**). Samples of the various strains were from stationary phase cultures that were serially diluted 1:10 repeatedly. They were spotted onto minimal media plates with the indicated concentrations of NaCl or LiCl. Plates were incubated for 72 hours at 30^o^ C. (**A**) Mutants E74A, R77A, E74R77A, E74R77RE, R341A, R342A, R341E, R342E. (**B**) Mutants NQQ, NQQQ, REL6, DEL6, E397L. KO, sod2:ura4 knockout; WT, wild type.

We then examined the ability of the wild type and these mutant *Sp*NHE1 proteins to restore salt tolerance in the sod2:ura4 strain, which has endogenous *Sp*NHE1 deleted^6^. Figure 2, A and B illustrate the salt tolerance in NaCl of the initial set of mutants (#1–9) and wild type and knockout strain. *S*. *pombe* with its own *Sp*NHE1 disrupted was able to grow robustly in liquid media with no added NaCl, and grew somewhat less robustly in media with 200 mM NaCl. There was not growth in 500 mM or 1 M NaCl. In contrast, *Sp*NHE1 containing wild type protein grew reasonably well in up to 500 mM NaCl. Of the first set of mutants (E74A, R77A, E74R77A, E74R77E, R341A, R342A, R341E and R342E), growth in NaCl containing liquid media was well maintained in the E74A and R77A mutants. Intermediate growth was maintained by the E74R77A mutant but less so in the remainder of the group. Similar results were obtained with growth on NaCl containing solid media (Fig. 3A).

The second group of mutants (NQQ, NQQQ, DEL6, E397L and REL6, #9–13) was examined in a similar fashion. The NQQ set of mutations did not affect NaCl tolerance in liquid media. In contrast, mutation of one more amino acid (Glu^397^ to Gln) caused a large change in the ability of *Sp*NHE1-NQQQ to confer salt tolerance (Fig. 2C). Notably, an individual mutation of Glu^397^ to Leu did not impair the ability of *Sp*NHE1 to confer salt tolerance. In the mutant DEL6, extracellular loop 6 (^390^EIEKSIYE^397^) was removed from the *Sp*NHE1 protein. This had an intermediate effect on the ability of the protein to confer salt tolerance.

Mutant number 13 REL6 replaced the residues ^390^EIEKSIYE^397^ of *Sp*NHE1 EL6 with SOS1-like residues from the protein SOS1 (QSSGNSHIKE). Surprisingly, this mutant functioned quite normally, conferring salt resistance to NaCl in liquid media (Fig. 2D).

Experiments examining the second group of mutants NaCl tolerance on solid media (Fig. 3B) were largely similar to those on liquid media, though they generally showed slightly more tolerance at low or intermediate NaCl concentrations (up to 500 mM). At high NaCl containing concentrations (1 M), growth was inhibited, including that of cells containing wild type *Sp*NHE1 (not shown).

LiCl tolerance of cells containing *Sp*NHE1 was also examined in both liquid and solid media (Fig. 1 supplementary, Fig. 3). When a mutation caused a lack of ability to confer NaCl tolerance, a similar defect occurred with LiCl (summarized in Table 1).

**Table 1:** Summary of growth of wild type or mutant yeast strains containing *Sp*NHE1 in liquid (L) or solid (S) media containing NaCl or LiCl. IDs identifying mutation type to amino acids are indicated.

Type	NaCl (L)	LiCl (L)	NaCl (S)	LiCl (S)	#
WT	+++	+++	+++	+++	
KO	−	−	−	−	
E74A	**+++**	+++	+++	++	1
R77A	**+++**	+++	++	++	2
E74R77A	**++**	+	+	+	3
E74R77RE	**+**	+	+	+	4
R341A	**+**	+	+	++	5
R342A	**+**	−	+	+	6
R341E	**+**	−	+	+	7
R342E	−	−	+	+	8
NQQ	**+++**	+++	+++	+++	9
NQQQ	−	+	+	+	10
DEL6	**+**	++	++	++	11
E397L	**+++**	+++	+++	+++	12
REL6	**+++**	+++	+++	+++	13

−, No growth; +, **++**, **+++**, increasing amount of growth with +++, indicating growth equivalent to wild type.

### SpNHE1 expression and localization

**Figure 4:**
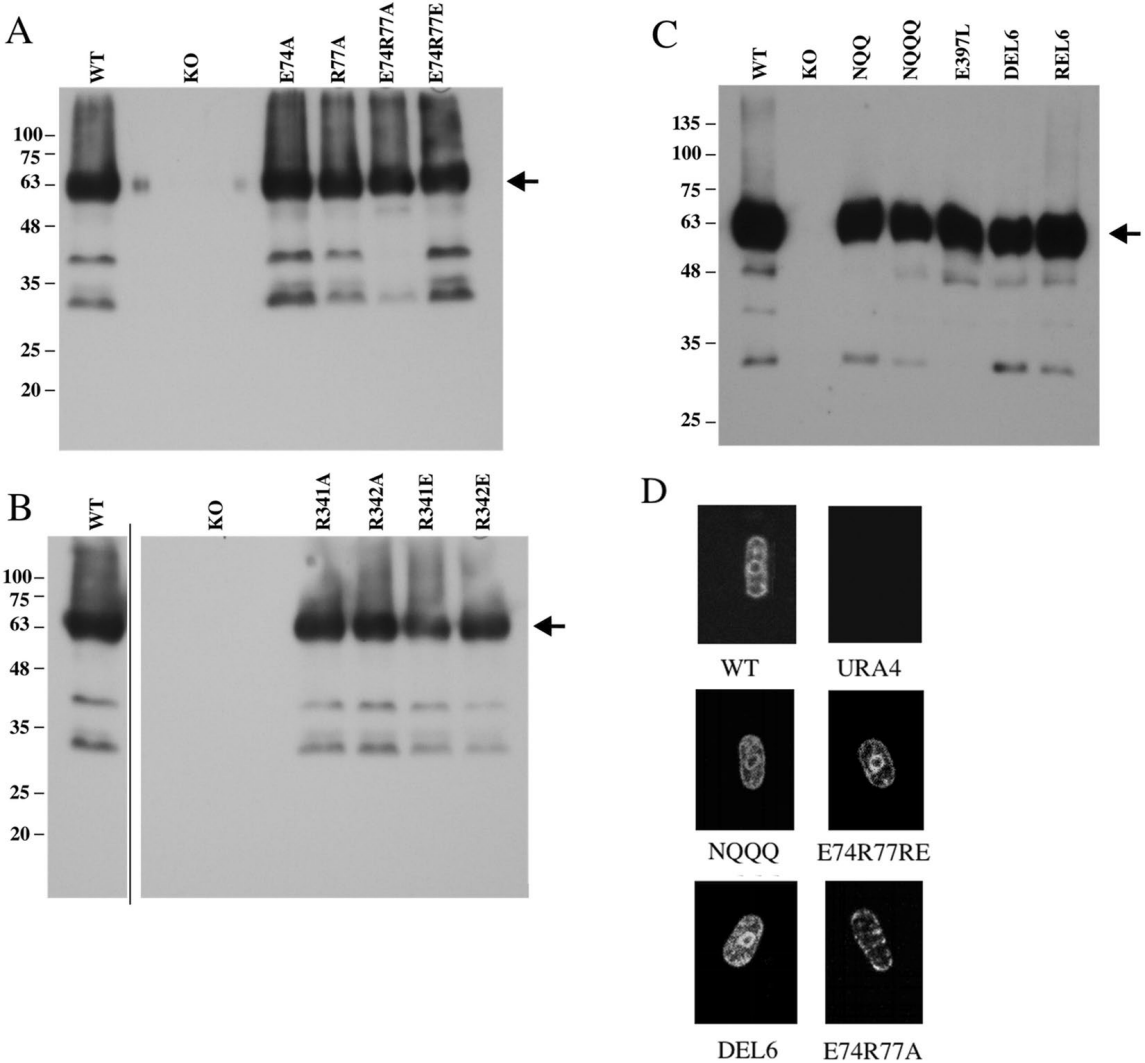
Expression and localization of wild type (WT) and mutant *Sp*NHE1. (**A–C**) Western blot analysis of expression of *Sp*NHE1 proteins from cell extracts of *S*. *pombe* strains expressing wild type or mutant *Sp*NHE1. The blot was immunostained with anti-GFP antibody. The arrow indicates the approximate location of the full-length protein (including the GFP tag). The mutations are indicated. (**D**) Confocal microscopy of wild type *Sp*NHE1, knock out (URA4) and selected *Sp*NHE1 mutants in *S*. *pombe*. Exponentially grown cells were harvested and used directly for live cell imaging of GFP fluorescence. (**B**) Solid line indicates where extraneous lanes were deleted. Left and right panels are from the same exposure of the same blot.

Western blot analysis against the C-terminal GFP tag was used to examine the expression of the mutant proteins (Fig. 4A–C). Wild type *Sp*NHE1 protein appeared as a major band of apparent molecular weight approximately 63 kDa. The knockout strain had no apparent protein while all the other mutants expressed full length *Sp*NHE1 protein. Only minor variations in the amount of full-length protein occurred. Figure 4D illustrates confocal microscopy of selected mutants which were defective in salt tolerance. All (except for the knockout strain) showed plasma membrane targeting of the GFP tagged *Sp*NHE1 protein. There was also a perinuclear staining in all the strains with *Sp*NHE1 except this was not shown in the E74R77A strain. We have earlier shown this type of staining, plasma membrane and perinuclear, in the wild type protein and in other mutants^6^. The results showed that targeting to the plasma membrane of several low activity mutants was similar to the wild type protein, which is what we have found earlier with most mutations of *Sp*NHE1^6,18^.

## Discussion

To examine the structural and functional aspects of the *Sp*NHE1 protein, a series of 13 different mutations to the protein were made. Mutants 1–4, involving Glu^74^ and Arg^77^, were initially thought to be located at the dimer interface Fig. 1C^17^ and we suspected they were involved in intermolecular electrostatic interactions. Therefore we mutated both residues to Ala (mutants 1 and 2). We also examined the effects of cumulative mutations (mutant #3) and we switched the two residues (mutant #4) as another way of examining if the two residues were involved with each other in an intermolecular interaction. Individual mutations of Glu^74^ to Ala (Table 1) and the Arg^77^ to Ala caused little reduction in the ability to confer salt tolerance. Mutation of both these residues to Ala caused larger reduction in function, as did switching the two residues. These results confirm that the residues are important in function of the protein but mutation of both, is required for a more notable effect. All the mutant proteins were expressed in levels similar to the wild type *Sp*NHE1 protein. Both these amino acids are conserved in the yeast species, but not so in the non-yeast Na^+^/H^+^ exchangers (Fig. 1A). In *E*. *coli* NhaA, the majority of the contact points of the interface of the dimer consists of two β-hairpins of the two monomers forming an antiparallel β-sheet at the periplasmic side of the molecule^19^. The dimer interface is amphipathic with alternating charged and non charged residues^20^. This kind of structure is not evident in the model of the *Sp*NHE1 protein^17^. Another Na^+^/H^+^ exchanger structure is that of NapA which lacks the β-hairpin domain^13^ as does NhaP1 of *Methanocaldococcus jannaschii*^15^ which contains an uncleaved signal sequence near the dimer interface. In NhaP1 it was suggested that the protomers are held together across an extensive contact surface at the dimer interface, which may be hydrophobic, in contrast to NhaA that has a liquid filled gap with minimal hydrophobic contacts^15^. NhaP1 showed little evidence of contact above or below the membrane^15^. UapA, a nucleobase ascorbate transporter is also a dimer with hydrophobic interactions mediating dimer formation^21^. Leucine zipper motifs have been suggested to be key in oligomerization of the dopamine transporter DAT and the GABA transporter GAT-1^21^. It therefore seems unlikely that Glu^74^ and Arg^77^ are involved in dimerization. As their mutation clearly affected function, it is more likely they have others roles to play. Charged amino acids within the membrane can have many different functions including affecting function by snorkeling^22^, interacting with phospholipids^23^ neutralizing helix dipoles^12^ and modulating helix orientation^24^. Structural analysis has suggested they can also form a salt bridge important in sodium and proton antiport^20^ and they have been shown to affect mammalian Na^+^/H^+^ exchanger function^25^.

While the role Glu^74^ and Arg^77^ are playing is not yet completely clear, and may await the elucidation of the structure of the protein, insights might be gained by comparison with other sequences and by examination of other proteins with known structures. Comparison of Na^+^/H^+^ antiporter sequences suggests that Glu^74^ and Arg^77^ are part of a conserved yeast plasma membrane motif EX_h_X_2_R where X_h_ is a hydrophobic residue and X_2_ is Ser, Thr, Cys or sometimes Ala (Fig. 1). TM 3 of *Pa*NhaP also contains a similar motif DXXR^14^ (Fig. 1). The sequence consists of Asp^59^ and Arg^62^ that are separated by Phe and Val. Examination of the *Pa*NhaP crystal structure suggests that Asp^59^ and Arg^62^ face the periplasmic side. As a consequence, they appear to induce a bend in TM 3 opening the periplasmic funnel of the protein. Arg^62^ is within interacting distance of the C-terminal of the TM 12, which is one of the discontinuous helices of the metal transport domain. Structural elucidations and mutational data suggested that the metal transport domain and the dimerization domain interact forming an elevator type of ion transport in Na^+^/H^+^ antiporters^26^. TM 3 belongs to the dimerization domain and TM 12 belongs to the metal transport domain. Therefore, Arg^59^ might bridge an interaction between the two domains. Supporting the hypothesis that TM 3 and TM 12 interact, are results that showed that the C-terminal residues of *Sp*NHE1 TM 12 are critical for proper function^6^. It is therefore possible that Arg^77^ mediates interactions with the transport domain however the structure of *Sp*NHE1 would need to be elucidated to confirm this hypothesis.

Two other amino acids that we examined in this study were Arg^341^ and Arg^342^. Part of our rationale for mutating these residues was that recent analyses of the sequence of Na^+^/H^+^ exchangers identified these two residues among several, yeast plasma membrane NHA- specific motifs^17^ (see also Fig. 1A). This “double-arginine” motif (RR) in putative segment TM 11 was particularly intriguing as it was conserved in the yeast species and one of the two Arg residues was conserved in other species. Here we demonstrated that it is essential for activity with both Arg^341^ and Arg^342^ necessary for transport in *S*. *pombe*. Mutation of either residue to Ala or negatively charged Glu, abolished or greatly reduced the ability of these proteins to confer salt tolerance on yeast. Arg^341^ and Arg^342^ are situated approximately in the middle of TM 11 and are the part of the larger motif “RRX_h_P”, where X_h_ is one of Leu, Ile, Val, or Met^17^. Sequence alignment of yeast PM-NHAs with NHAs from other domains, shows that the corresponding position in TM11 (TM10 for *Ec*NhaA) of bacteria, archaebacteria, plant, and mammals contains one basic residue (Fig. 1A). The role of this residue in function may vary between Na^+^/H^+^ antiporter members, or is subject to controversy. Mutational data, kinetics analysis and molecular dynamics simulation suggested that this basic residue Lys^305^ is essential for electrogenic transport of cations, and participates in cation binding in NapA^27,28^. However, another study has proposed an indirect involvement of the corresponding Lys300 of NhaA, providing both a structural and functional role, but not an exclusive requirement for electrogenic ion transfer^29^. It should be noted that in both cases the basic residue is important for efficient ion transport as well as the thermal stability.

We were curious as to why yeast species have a basic residue pair and other species do not. We examined the structures and sequence of several other Na^+^/H^+^ antiporters. We note that except for the *E*. *coli*, another basic residue (often arginine) is present in transmembrane segment 12 of many species (Fig. 1B and ^17^) including in *Tth*NapA^13^, *Mj*NhaP1^30^, and *Pa*NhaP^14^ (Arg^362^ in PaNhaP, Arg^347^ in *Mj*NhaP1, and Arg^331^ in *Tth*NapA). Examination of the atomic resolution structures of these Na^+^/H^+^ antiporters suggests that these Arg side chains are involved in salt bridge interactions with the polypeptide backbone. A critical feature of Na^+^/H^+^ exchangers, of the cation proton antiporter family for which structures have been deduced, is that they posses a fold consisting of two crossed helices with a discontinuous non helical extended segment mid membrane^12^. The salt bridge interaction of these Arg residues is in vicinity of the helical discontinuous region of these transmembrane segments. It is possible that the Arg-backbone salt bridge interaction provides structural constraints to the helical discontinuity/ flexibility. The structure of *Sp*NHE1 has not yet been elucidated so the exact role of Arg^342^ in maintaining *Sp*NHE1 structure and function is still to be determined. It may be that in yeast, the number of Arg residues is finely tuned. Introduction of an additional Arg within transmembrane segments of *Sp*NHE1 can result in an inactive or severely defective protein^6^. In species other then yeast, the arginines are present in two different helices. One Arg may provide the structural constraints and the other may provide a proper environment for metal transport. In yeast such as *Sp*NHE1, there is no Arg residue in predicted TM12^17^ and these two arginines are clustered in the same TM segment.

We have earlier^31^ suggested that binding of cations could be based on coordination of the cations by a crown ether-like cluster of polar amino acids as originally hypothesized by Boyer^32^. Additionally, several extracellular and intracellular loops of the human NHE1 isoform of the Na^+^/H^+^ exchanger have been shown to be important in activity of the protein^33–35^. When multiple sequence analysis was done between Na^+^/H^+^ antiporters (Fig. 1, and^17^), one yeast specific loop emerged that is rich in Asp and Glu residues. Based on the predicted topology of *Sp*NHE1, that loop was designated as extracellular loop 6 (EL6) that joins TM12 to TM13^17^. In general, EL6 is variable in length between plasma membrane Na^+^/H^+^ antiporters but is more elongated in the yeast group compared with NHAs from bacteria, plant and mammalian groups. EL6 of *Sp*NHE1 contains the polar residues Asp^389^, Glu^390^, Glu^392^, and Glu^397^ and molecular modeling^17^ suggests Asp^389^, Glu^390^ and Glu^392^ are more towards TM12, while Glu^397^ may be more towards the middle of the loop (Fig. 1D). Previously^6^, Ala scanning mutagenesis showed that the individual residues Asp^389^, Glu^390^ and Glu^392^ are not critical to protein function. Mutation number 9 (NQQ) neutralized the charges of all 3 residues to similar sized and uncharged amino acids but only had a very minor effect on function of the protein. The NQQ mutant was used as a template to create mutation number 10 (NQQQ), with the additional E397Q mutation. This resulted in a mutant protein that was not able to confer salt tolerance. This was likely not due to deletion of E397Q itself, but was a cumulative effect of the replacements of Asp^389^, Glu^390^, Glu^392^, and Glu^397^. Deletion of EL6 amino acids ^390^EIEKSIYE^397^ (mutant #11, DEL6) had only a minor effect on the ability to confer salt tolerance and this mutation removed the amino acids Glu^390^ and Glu^392^ and Glu^397^. Additionally, mutation E397L alone did not have a large effect on the ability of the protein to confer salt tolerance. It thus seems that there is a requirement for negatively charged residues in this region, but each individual residue is not absolutely required. Again, confocal images of NQQQ show that the mutation did not hinder the localization to the plasma membrane. The inactivity of the NQQQ mutant is thus due to compromised activity.

We also examined if the EL6 residues are replaceable with a corresponding region of the plant plasma membrane Na^+^/H^+^ antiporter SOS1 (mutant 13, REL6). We replaced the residues ^390^EIEKSIYE^397^ of *Sp*NHE1 with plant Na^+^/H^+^ antiporter SOS1-like residues from the protein of *Arabidopsis thaliana* (QSSGNSHIKE). The plant Na^+^/H^+^ antiporter was chosen because the principal function of both *Sp*NHE1 and SOS1 is to remove Na^+^ to the outside of the cell. The replacement of these amino acids had no affect on the ability of the protein to confer salt tolerance. These results are in agreement with the suggestion that acidic residues are required in this region, but an absolute position is not strictly required. One or two acidic amino acids maintain protein function. Here, we returned one Glu in the replacement sequence and amino acid Asp^389^ was retained. This would be in agreement with our results that showed that deletion of residues ^390^EIEKSIYE^397^, which maintains one acidic amino acid, Asp^389^, still maintains *Sp*NHE1 function. There was a slight effect on conveyance of salt tolerance with reducing the acidic residues to a single amino acid whereas when two acidic residues remained, with the replacement of the fragment with the SOS1-like Asp containing sequence, function was completely maintained. Overall, it seems that the region is not highly specific for the location of acidic charges, but requires one and ideally two acidic amino acids to maintain function. We suggest that this negatively charged density due to the acidic residues, may be acting to aid in attraction of cations to the membrane protein pore. A cation or ion coordination sphere has been demonstrated in other membrane proteins, including the acetylcholine receptor^36,37^ and NhaA^12^.

Overall, our results have determined that EL6 plays an important role in the ability of the *Sp*NHE1 protein to confer salt tolerance in yeast. We also found that *Sp*NHE1 Arg^341^ and Arg^342^ are necessary for *Sp*NHE1 transport in *S*. *pombe* and that Glu^74^ and Arg^77^ form an important, conserved, yeast motif of *Sp*NHE1 that may mediate intramolecular interactions. In the present study, we used our earlier predicted model of *Sp*NHE1^17^. *Pa*NhaP is the Na^+^/H^+^ antiporter of *Pyrococcus abyssi* that has highest sequence identity (21%) with *Sp*NHE1^17^. In addition, the secondary structure predictions of *Sp*NHE1 also match the *Pa*NhaP secondary structure and hence we reasoned that among the existing three-dimensional structures of Na^+^/H^+^ antiporters, the *Sp*NHE1 structure could best be compared with *Pa*NhaP. Future studies will determine if the crystal or cryo-electron microscope structure of *Sp*NHE1 corresponds with our predicted structure of the protein.

## Materials and Methods

### Materials

Synthetic DNA for site-specific mutagenesis was from Integrated DNA Technologies. Restriction enzymes were obtained from New England Biolabs, Inc. or *In vitrogen*. PWO DNA polymerase for DNA amplification was from Roche Applied Science (Roche Molecular Biochemicals, Mannheim, Germany). All other chemicals were of analytical grade and were purchased from Fisher Scientific (Ottawa, ON), Sigma or BDH (Toronto, ON).

### Strains and media

*S*. *pombe* with the *SpNHE1* gene disrupted (previously referred to as sod2::ura4) was used as to reintroduce wild type and mutant forms of the *Sp*NHE1 protein^6,8^. The knock out strain was maintained on sodium deficient minimal KMA medium or yeast extract adenine (YEA) as described earlier^7,8^. KMA medium consists of K_2_HPO_4_, 3 g; potassium hydrogen phthalate, 3 g; yeast nitrogen base without amino acids, 7 g; glucose, 20 g; and adenine, 200 mg (per 1 liter). Leucine at 200 mg/l was added when necessary to maintain the *sod2::ura4 leu1–32* strain as indicated. All media was buffered using 50 mM MES/Citrate and pH adjusted to 5.0 with KOH. Wherever indicted LiCl or NaCl were added to media at the concentrations indicated. pREP-41sod2GFP contains a full-length *Sp*NHE1 gene plus a C-terminal GFP tag, which is separated from *Sp*NHE1 by a nine amino acid (Gly-Ala) spacer. The GFP protein has the Ser65Thr mutation. An NdeI site was removed by silent mutation to aid in cloning. This plasmid, pREP-41sod2GFP was used to express the *Sp*NHE1 protein with a fused GFP protein^6,38^.

Transformation of the plasmids with and without mutations, was by electroporation into the *Sp*NHE1 knock out strain sod2::ura 4^39^. Briefly, cells were grown with vigorous shaking in 100 ml of KMA (plus Leucine) media at 30 °C until an OD600 of 0.5 to 1.2. The cells were then incubated on ice for 15 min and harvested by centrifuging at 3500 × g × 5 min at 4 °C. The pellet was resuspended in 200 ml ice-cold water. Washed cells were collected by centrifugation (3500 × g × 5 min) and washed further with 50 ml of ice-cold 1.0 M sorbitol two times. The cell pellet was resuspended finally in 1 ml of 1.0 M sorbitol. This was divided into 200 μL of aliquots and mixed with 0.1 ng of purified DNA. A cell-DNA mixture was transferred to a cuvette for electroporation (0.2 cm) pre-incubated in ice. After 5 min an electric pulse was applied according with a Gene Pulser II (Bio-Rad). Cells were immediately resuspended in 800 μL of 1.0 M sorbitol and incubated at 30 °C for 1 hr without shaking. Cells were harvested by centrifugation and spread onto KMA agar containing 1.0 M sorbitol without leucine for selection and growth over 3–4 days.

Growth of strains containing the pREP-41sod2GFP plasmid was in liquid and on solid media. For liquid growth 5 × 10^6^ cells were taken from an overnight exponentially growing culture and inoculated into 2.5 ml of fresh liquid media liquid media. Transformed *S*. *pombe* were routinely grown in medium in the absence of thiamine. Cultures were grown with constant agitation at 30 °C in a rotary shaker. The optical density at 600 nm was determined at the indicated times. Growth curves were determined at least three times and results are the mean +/− SE.

To examine growth on plates, serial dilutions of cells were inoculated onto agar with KMA medium containing leucine supplemented with either NaCl or LiCl at the indicated concentrations. The control pREP-41sod2GFP plasmid containing *Sp*NHE1 without mutations^38^ was used as a control.

### Site-directed mutagenesis

Mutations to the pREP-41sod2GFP plasmid containing *Sp*NHE1 was by PCR amplification using synthetic oligonucleotides (See Supplementary Table 1).

Most mutations created or removed a restriction enzyme site^40^. A total of 13 types of mutations were done. DNA sequencing confirmed the accuracy of the mutations and the fidelity of DNA amplification. Briefly, for type 1 and 2 mutations Glu^74^ and Arg^77^ were individually changed to Ala using the primers described (E74A and R77A respectively) (See Supplementary Table 1). To examine the cumulative effect of mutation of both to Ala, the primer set E74R77A was used (#3). Another mutation was done to exchange to position of the two amino acids, the primer set E74R77RE was used (#4). To investigate the roles of Arg^341^ and Arg^342^ initially each were individually mutated to Ala (R341A and R342A primers, #5, #6) and then later the charge was changed from positive to negative (Glu) with the primer set R341E and R342E (#7, #8). Asp389, Glu390, Glu392, and Glu397 are on putative extracellular loop six (discussed below). To examine their importance in the protein first we converted Asp^389^, Glu^390^, Glu^392^, to their corresponding amide counterparts using the primer set D389N,E390Q,E392Q. The resulting mutant was designated as DEE (or NQQ, #9). The NQQ mutant was used as a template to mutated Glu^397^ to Gln with the primer set E397Q. This yielded a four amino acid mutant called DEEE (or NQQQ, #10). To further examine this region we deleted amino acids ^390^EIEKSIYE^397^ and the primer set delEL6 was used for this purpose (#11, DEL6). Additionally, to examine the role of Glu^397^ in further detail we made the mutation E397L (#12).

For mutation number 13 we replaced the residues ^390^EIEKSIYE^397^ of *Sp*NHE1 with SOS1-like residues from the protein SOS1 (QSSGNSHIKE). The modified sequence was chosen such that it contains at least one acidic residue as well as roughly the same amino acid length that of the deleted sequence. To do this we amplified *Sp*NHE1 in two parts using the primer sets ReEL6SOS2FP/ReEL6SOS2RP and ReEL6SOS4FP/ReEL6SOS3RP. The amplified products were used in overlap-extension PCR using ReEL6SOS2FP/ReEL6SOS3RP primers.

### Western blotting analysis

Western blot analysis of *SpNHE1* compared the levels of *Sp*NHE1 protein expression in cell lysates from wild type and mutant *Sp*NHE1 containing yeast^6,40^. Cell lysates were made from transformed yeast cultures grown as described above. The cultures were pelleted at 3500 x g, 10 min, and washed with double distilled water. They were then resuspended in lysis buffer consisting of 50 mM Tris-HCl, pH 8.0, 5 mM EDTA, 1 mM dithiothreitol containing a protease inhibitor cocktail^41^. Cells were lysed using a Bullet Blender^®^ with 0.5 mm zirconium oxide beads at a speed of 10 × 40 minutes. Alternatively, they were lysed by passage through an emulsiflex homogenizer with a pressure of 25000 psi. Unlysed cells were pelleted via centrifugation at 3500 × g for 5 min, and the supernatant was centrifuged (14000 × g × 10 min). Enriched membranes present in the supernatant were pelleted at 100000 × g for 1 hr. These were resuspended in a small volume of 50 mM Tris-Cl pH 8.0, 150 mM NaCl, 5 mM EDTA, 1 mM EGTA, 1.0% (v/v) NP-40, 0.5% (w/v) deoxycholate and 0.1% (w/v) SDS. Equal amounts of samples of up to 25 μg were resolved on SDS/polyacrylamide gels (10%). Western blotting of nitrocellulose transfers used an anti-GFP polyclonal antibody (a generous gift of Dr. Luc Berthiaume, Dept. of Cell Biology, University of Alberta). Secondary antibody was goat anti-mouse antibody peroxidase-conjugated (Bio-Can, Mississauga, Canada). X-ray film detected protein reactivity and was via the Amersham enhanced chemiluminescence western blotting and detection system.

### SpNHE1 sequence alignment

*Sp*NHE1 multiple sequence alignment was performed using MAFFT^42^. The alignment was prepared using ESpript^43^.

### Microscopy and indirect immunofluorescence

Confocal imaging was used to examine localization of *S*. *pombe* containing GFP tagged *Sp*NHE1. This was performed on an Olympus IX81 microscope equipped with a spinning-disk optimized by Quorum Technologies (Guelph, ON, Canada). Images were acquired using Volocity software (Improvision Inc., Lexington, MA) with a 60 X objective on a Hamamatsu EM-CCD camera (Hamamatsu, Japan). Yeast cells were either immobilized with 1% gelatin or for live cell imaging of the GFP tag, confocal microscopy was essentially as described earlier^38^.

## Supplementary information

SUPPLEMENTARY Dataset 1
