# Supplementary material for: Functional Analysis of Conserved Transmembrane Charged Residues and a Yeast Specific Extracellular Loop of the Plasma Membrane Na+/H+ Antiporter of Schizosaccharomyces pombe
Source: Sci Rep. 2019 Apr 17;9:6191. doi: 10.1038/s41598-019-42658-0 (PMC6470128; doi:10.1038/s41598-019-42658-0)
Supplement: Supplementary file 1 — SUPPLEMENTARY Dataset 1 [file 41598_2019_42658_MOESM1_ESM.pdf]

Functional Analysis of Conserved Transmembrane Charged Residues and a Yeast Specific Extracellular Loop of the Plasma Membrane Na^+/H^+ Antiporter of *Schizosaccharomyces pombe*

Debajyoti Dutta[1], Asad Ullah, Sana Bibi and Larry Fliegel[1]

[1] *Department of Biochemistry, University of Alberta, Edmonton, Alberta, Canada T6G 2H7.*

Supplementary Material

Supplementary Table 1. Oligonucleotide primers for site-directed mutagenesis. Lower case indicates altered base pair. Complementary oligonucleotides were synthesized but are not indicated.

Mutation	Sequence	Restriction Site	#	ID
E74Af	5′-GGAGATTACTTGACAGTgGccATTTGTAGAATCGTACTTGATGTG-3′	*MscI*	1	E74A
R77Af	5′-CACATCAAGTACGATTCTACAAATggCcACTGTCAAGTAATCTCC-3′	*PvuI*	2	R77A
E74R77Af2	5′-GATTACTTGACAGTAGccATaTGTgcAATCGTACTTGATGTG-3′	*NdeI*	3	E74R77A
E74R77REf	5′-CCATGGAGATTACTTGACAGTgcgcATTTGTgaAATCGTACTTGATGTG CGTGTG-3′	*FspI*	4	E74R77RE
R341Af	5′-CTTTAGCATATTGACTCTAGTTTGcgcaCGATTACCGGTTGTATTTTCG-3′	*FspI*	5	R341A
R342Af	5′-GCATATTGACTCTAGTTTGTCGagctcTACCGGTTGTATTTTCGGTG-3′	*SacI*	6	R342A
R341Ef	5′-GCATATTGACTCTAGTTTGTg<u>agCGcT</u>TACCGGTTGTATTTTCGG-3′	*AfeI*	7	R341E
R342Ef	5′-GCATATTGACTCTAGTTTGTCGTgagcTcCCGGTTGTATTTTCGGTGAAG-3′		8	R342E
D389N, E390Q, E392Q	5′-GGCATTTCTTGCAAAATTACTcTTaagCCCGaATcAAATTcAAAAGAGTAT TTATGAATCAAC-3′	*AflII*	9	NQQ
E397Q	5′-CAAAAGAGTATTTATcAgTCgACTACAGTATTTTCAAC-3′	*Sal1*	10	NQQQ
DelEL6	5′-GTTTATATGGCATTTCTTGCAAAgcTtCTGTTGTCCCCGGATTCAACTACA GTATTTTCAACACTAAATG-3′	*HindIII*	11	DEL6
E397L	5′-GAAAAGAGTATTTATctgTCgACTACAGTATTTTCAACAC-3′	*Sal1*	12	E397L
ReEL6SOS2FP	5′-GTTAAATcatATGGGCTGGAGACAACTTGATATAG-3′		13	
ReEL6SOS2RP	5′-CTTCCTTATTATGAgaATTcCCGAAACTTTGttttgcaagaaatgccatataa actgcgcaaac-3′		13	
ReEL6SOS4FP	5′-CAAAGTTTCGGgAATtcTCATAATAAGGAAgtattttcaacactaaatgaa ataatttgg-3′		13	
ReEL6SOS3RP	5′-gcGggatccggaacgtaatcttcctgtgacttaTC-3′		13	REL6

Fig. 1Supplementary. Growth of *S. pombe* containing either wild type or mutant *Sp*NHE1 proteins in liquid media with concentrations of LiCl of 0, 5 mM, 10 mM or 20 mM. Results are the mean +/- SE of a minimum of three determinations. **A**, **B,** comparison of growth rates in LiCl medium of control and mutant strains. **C, D** second round of mutagenesis to other amino acids or segments of *Sp*NHE1. Ura4 refers to *S. pombe* with the *Sp*NHE1 knockout described in "Materials and Methods".

A

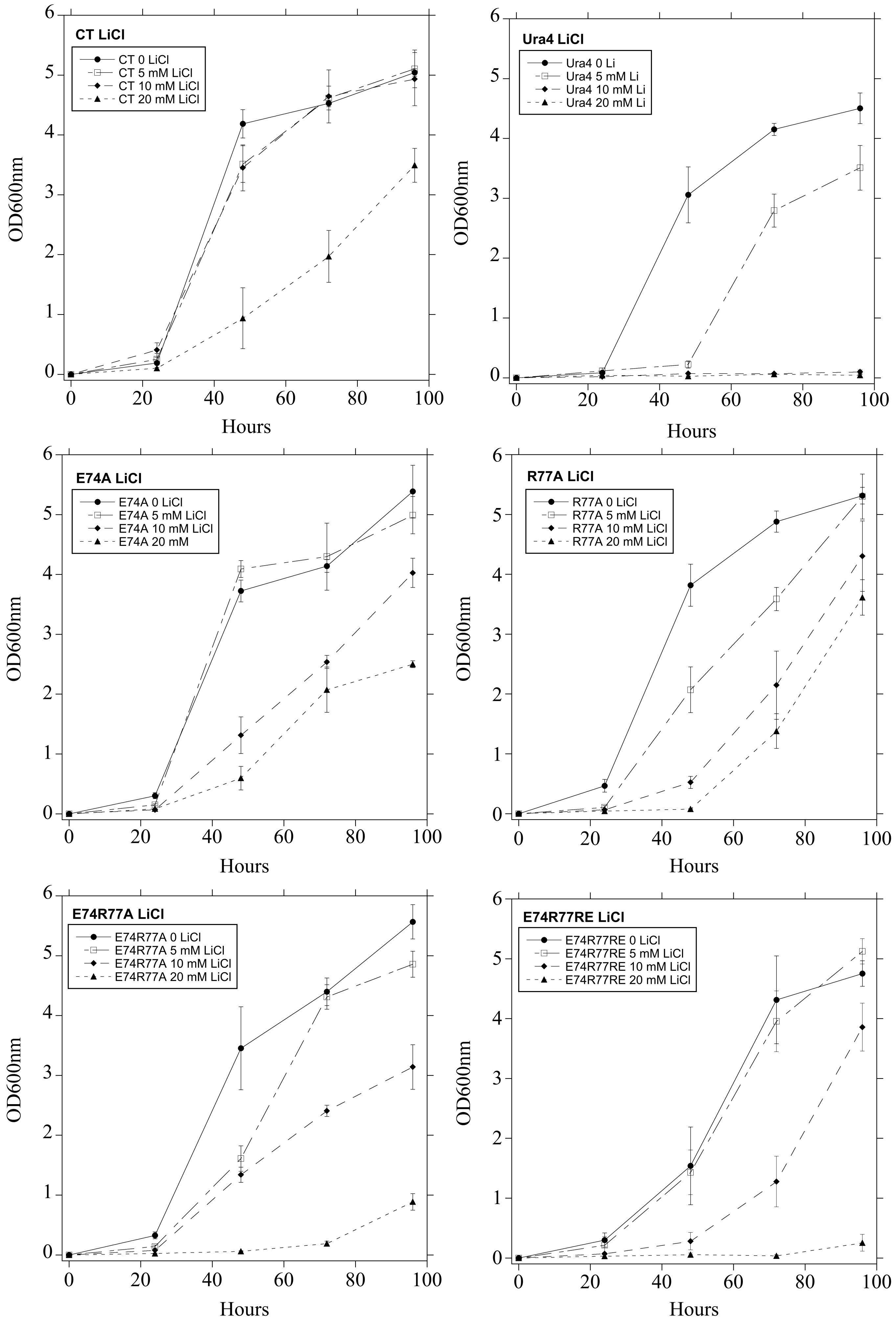
CT LiCl
CT 0 LiCl
CT 5 mM LiCl
CT 10 mM LiCl
CT 20 mM LiCl
Ura4 LiCl
Ura4 0 Li
Ura4 5 mM Li
Ura4 10 mM Li
Ura4 20 mM Li
E74A LiCl
E74A 0 LiCl
E74A 5 mM LiCl
E74A 10 mM LiCl
E74A 20 mM
R77A LiCl
R77A 0 LiCl
R77A 5 mM LiCl
R77A 10 mM LiCl
R77A 20 mM LiCl
E74R77A LiCl
E74R77A 0 LiCl
E74R77A 5 mM LiCl
E74R77A 10 mM LiCl
E74R77A 20 mM LiCl
E74R77RE LiCl
E74R77RE 0 LiCl
E74R77RE 5 mM LiCl
E74R77RE 10 mM LiCl
E74R77RE 20 mM LiCl
OD600nm
Hours
0
1
2
3
4
5
6
0
20
40
60
80
100

B

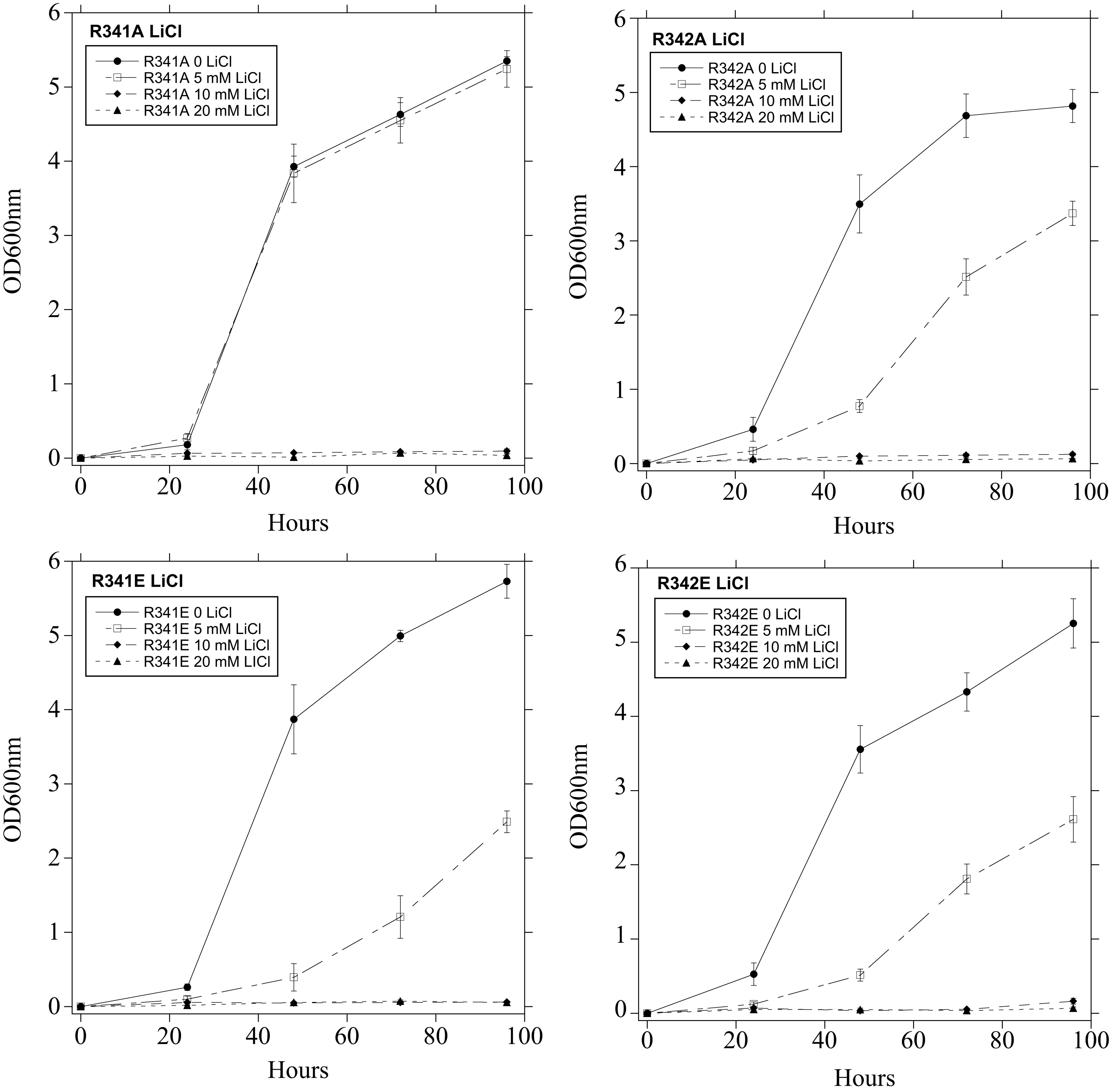
R341A LiCl
R341A 0 LiCl
R341A 5 mM LiCl
R341A 10 mM LiCl
R341A 20 mM LiCl
R342A LiCl
R342A 0 LiCl
R342A 5 mM LiCl
R342A 10 mM LiCl
R342A 20 mM LiCl
R341E LiCl
R341E 0 LiCl
R341E 5 mM LiCl
R341E 10 mM LiCl
R341E 20 mM LiCl
R342E LiCl
R342E 0 LiCl
R342E 5 mM LiCl
R342E 10 mM LiCl
R342E 20 mM LiCl
OD600nm
Hours

C

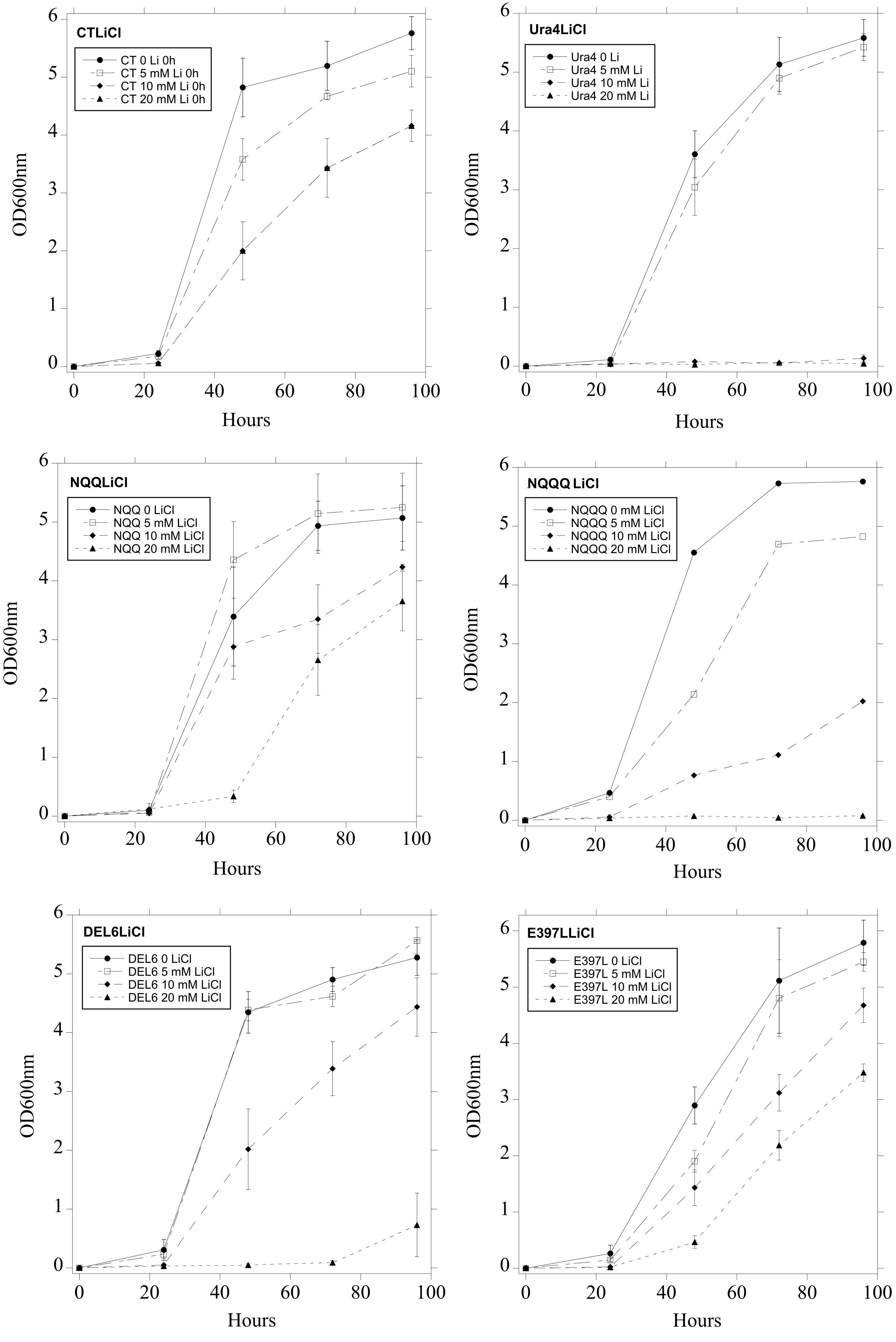
CTLiCl
CT 0 Li 0h
CT 5 mM Li 0h
CT 10 mM Li 0h
CT 20 mM Li 0h
OD600nm
Hours
Ura4LiCl
Ura4 0 Li
Ura4 5 mM Li
Ura4 10 mM Li
Ura4 20 mM Li
OD600nm
Hours
NQQLiCl
NQQ 0 LiCl
NQQ 5 mM LiCl
NQQ 10 mM LiCl
NQQ 20 mM LiCl
OD600nm
Hours
NQQQ LiCl
NQQQ 0 mM LiCl
NQQQ 5 mM LiCl
NQQQ 10 mM LiCl
NQQQ 20 mM LiCl
OD600nm
Hours
DEL6LiCl
DEL6 0 LiCl
DEL6 5 mM LiCl
DEL6 10 mM LiCl
DEL6 20 mM LiCl
OD600nm
Hours
E397LLiCl
E397L 0 LiCl
E397L 5 mM LiCl
E397L 10 mM LiCl
E397L 20 mM LiCl
OD600nm
Hours

D

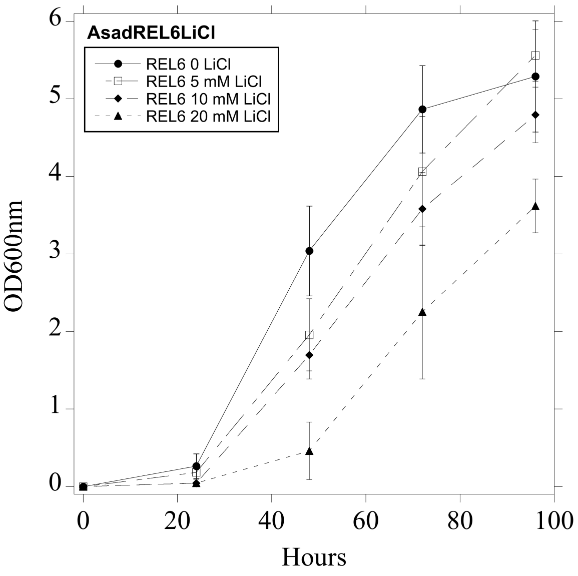
AsadREL6LiCl
REL6 0 LiCl
REL6 5 mM LiCl
REL6 10 mM LiCl
REL6 20 mM LiCl
OD600nm
Hours
0
1
2
3
4
5
6
0
20
40
60
80
100
